# Graphene: An Antibacterial Agent or a Promoter of Bacterial Proliferation?

**DOI:** 10.1016/j.isci.2020.101787

**Published:** 2020-11-11

**Authors:** Tian Zhang, Pier-Luc Tremblay

**Affiliations:** 1 State Key Laboratory of Silicate Materials for Architectures, Wuhan University of Technology, Wuhan 430070, PR China; 2 School of Chemistry, Chemical Engineering and Life Science, Wuhan University of Technology, Wuhan 430070, PR China; 3 School of Materials Science and Engineering, Wuhan University of Technology, Wuhan 430070, PR China

**Keywords:** Surface Property, Microbiology, Microbiofilms

## Abstract

Graphene materials (GMs) are being investigated for multiple microbiological applications because of their unique physicochemical characteristics including high electrical conductivity, large specific surface area, and robust mechanical strength. In the last decade, studies on the interaction of GMs with bacterial cells appear conflicting. On one side, GMs have been developed to promote the proliferation of electroactive bacteria on the surface of electrodes in bioelectrochemical systems or to accelerate interspecies electron transfer during anaerobic digestion. On the other side, GMs with antibacterial properties have been synthesized to prevent biofilm formation on membranes for water treatment, on medical equipment, and on tissue engineering scaffolds. In this review, we discuss the mechanisms and factors determining the positive or negative impact of GMs on bacteria. Furthermore, we examine the bacterial growth-promoting and antibacterial applications of GMs and debate their practicability.

## Graphical Abstract

**Figure:**
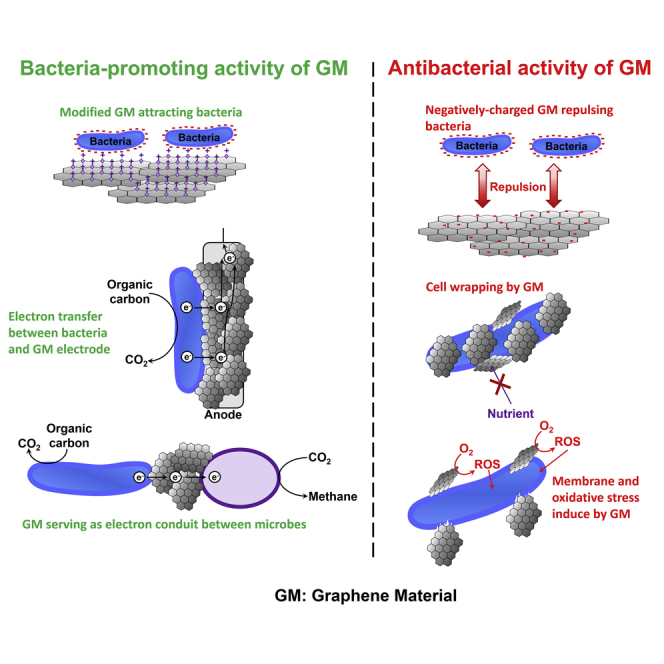

## Introduction

In the last decade, graphene materials (GMs) have been extensively studied for multiple applications in the environmental, electrochemical, and medical fields (Novoselov et al., 2004; ElMekawy et al., 2017; Zou et al., 2016). The vast research interest into GMs is born from its unique set of characteristics including excellent electrical conductivity, inherent mechanical strength, lightness, outstanding specific surface area (SSA), and high thermal conductivity (Perreault et al., 2015a; McAllister et al., 2007; Geim and Novoselov, 2007). Another important aspect of GMs for industrial usages is that it is easier to synthesize with a lower fabrication cost than other nanocarbons such as carbon nanotubes (CNTs) (Pumera, 2009; ElMekawy et al., 2017). Furthermore, GMs can readily be functionalized or combined with other materials into composites for a wide range of purposes (Hegab et al., 2016; Xia et al., 2019).

Because of its physicochemical properties, research groups have investigated the potential of GMs for a plethora of biotechnologies (Tremblay et al., 2020; Zhang et al., 2011b; Ji et al., 2016; Mohammed et al., 2020). The study of the interactions of GMs with living cells and more specifically with bacteria has led to discoveries that appear conflicting. On the one hand, multiple reports describe GMs promoting bacterial growth and biofilm formation (ElMekawy et al., 2017; Ruiz et al., 2011; Hegab et al., 2016). In these studies, pristine graphene (Gr), graphene oxide (GO), and reduced graphene oxide (rGO) are put forward as high-performance materials for diverse applications where bacterial metabolism must be stimulated such as power generation by microbial fuel cells (MFCs) or the acceleration of methane production by anaerobic digestion (Cotts et al., 2020; Tian et al., 2017). On the other hand, a large body of work details GMs that damage bacterial cells and inhibit growth (Rojas-Andrade et al., 2017; Kumar et al., 2019). Antibacterial GMs have potential usages in the medical and environmental fields for applications where the formation of pathogenic or fouling biofilms must be prevented (Kumar and Chatterjee, 2016; Wang et al., 2019a; Firouzjaei et al., 2020; Xia et al., 2019).

Numerous questions remained on why GMs can exhibit both bacterial growth-promoting activity and antibacterial activity. In this review, we discuss the factors and mechanisms governing the interactions of GMs with bacteria. Furthermore, we present and debate the merits of the different environmental and medical applications of antibacterial GMs and GMs beneficial for bacteria.

## Types of Graphene

**Figure 1:**
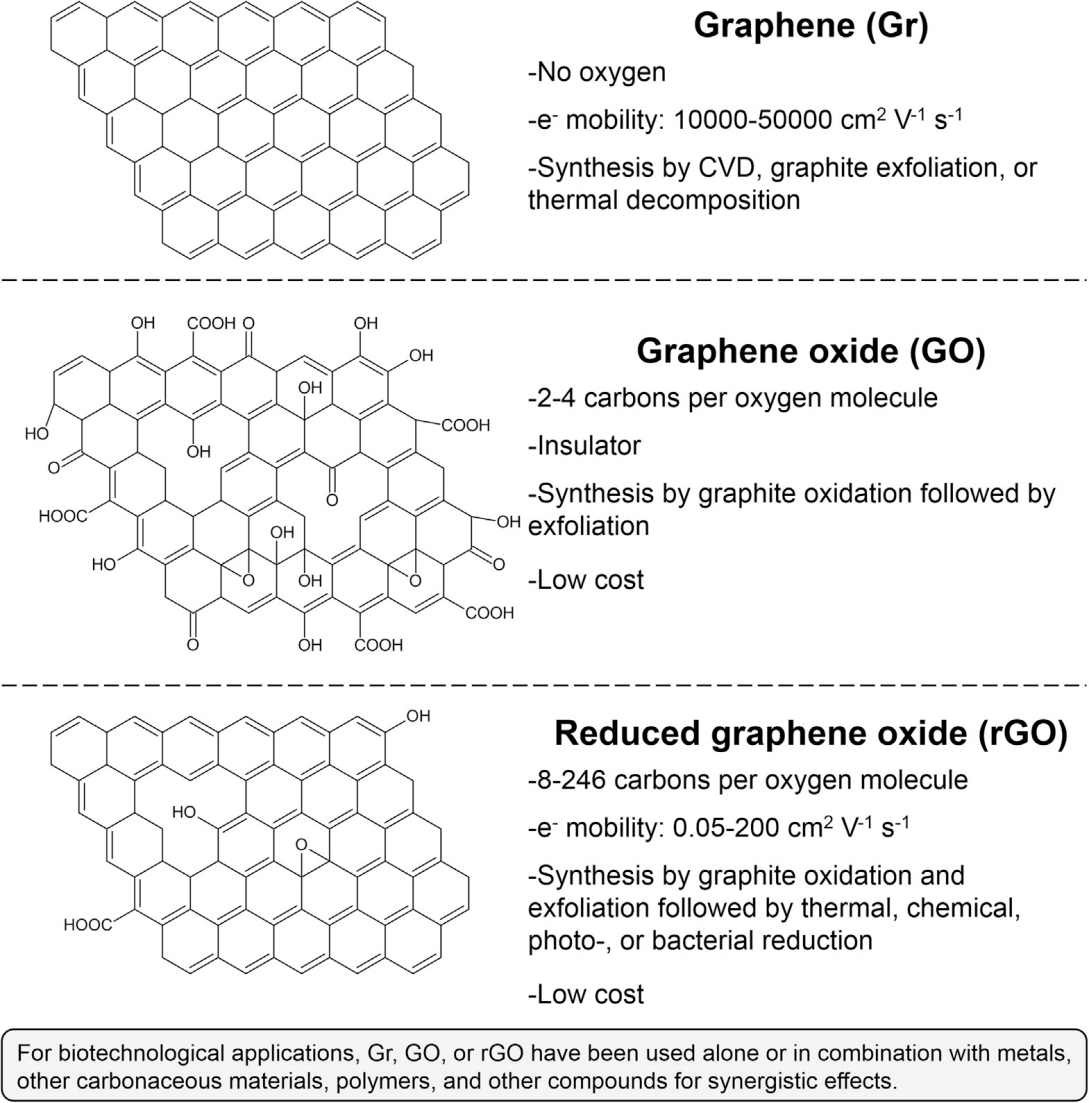
Structure and General Properties of Gr, GO, and rGO for Bacteria-Related Applications Oxygen composition, electron mobility value, and synthesis methods are reviewed in Perreault et al. (2015a).

Graphene materials studied for biocompatible or antibacterial applications can be divided into three main types according to their physicochemical properties: Gr, GO, and rGO (Figure 1) (Perreault et al., 2015a). Graphene is made of carbons arranged in a single layer of sp^2^ aromatic ring. It can be generated from graphite exfoliation, by thermal decomposition of silicon carbide, or via chemical vapor deposition (CVD) on metal substrates (Geim, 2009; Geim and Novoselov, 2007; Zhu et al., 2010; de Heer et al., 2007; Li et al., 2009; Novoselov et al., 2004). The specifics, advantages, and disadvantages of the three synthesis methods for Gr have been extensively reviewed by Lee et al. (2017) and by Backes et al., 2020, among others (Choi et al., 2010; Lee et al., 2017). Graphene has excellent electron mobility and mechanical strength, and because it is a single-plane material exposed on both sides, it has extremely high SSA (Bolotin et al., 2008; Sreeprasad and Berry, 2013; Lee et al., 2008; Sanchez et al., 2012). Because of these characteristics, Gr has been widely studied and developed in the last two decades as a promising material for multiple electronic applications including bioelectrochemical systems (BESs) (Randviir et al., 2014; Aïssa et al., 2015; Guirguis et al., 2020; ElMekawy et al., 2017).

Graphene oxide is produced at a lower cost than Gr by chemically oxidizing graphite to graphite oxide followed by an ultrasonication step to separate the multiple layers of graphite oxide (Figure 1) (Dreyer et al., 2009; Hummers and Offeman, 1958). The purpose of the oxidation step is to augment the distance between the multiple layers of graphite and reduce the energy required for the exfoliation process (Zhu et al., 2010; Lerf et al., 2006). Graphene oxide has a similar structure than Gr but with multiple carboxyl, epoxy, carbonyl, and hydroxyl functional groups. These groups in the graphemic structure reduce significantly electron mobility and mechanical strength but augment hydrophilicity (Sreeprasad and Berry, 2013; Perreault et al., 2015a; Dreyer et al., 2009).

Reduced graphene oxide is generated by the reduction of GO via multiple methods such as thermal annealing, chemical reducing agents, photoreduction, and bacterial reduction (Figure 1) (Hegab et al., 2016; Chua and Pumera, 2013; Pei and Cheng, 2012). The conversion of GO by these approaches in rGO significantly reduces the oxygen content but does not result in pristine Gr (Pei and Cheng, 2012). Reduced graphene oxide exhibits residual oxygen functional groups, carbon vacancies, and irregular carbon lattice structures not found in pristine Gr (Gómez-Navarro et al., 2010; Bagri et al., 2010). Consequently, rGO possesses better mechanical strength than GO as well as better electron mobility that varies according to the level of reduction. However, both the electron mobility and the mechanical strength of rGO are not as high as what is observed with pristine Gr.

Because of these physical, chemical, structural, and electronic differences, Gr, GO, and rGO will interact differently with bacteria. For BESs, the electronic properties and large SSA of Gr and rGO are particularly advantageous for the fabrication of performant electrodes because they ensure efficient electron transfer with a maximum number of microbial cells (Tremblay et al., 2020). On the other side, reports that may seem somewhat conflicting suggest that Gr or rGO are more efficient at inhibiting bacterial growth than GO because of higher electronic conductivity translating into a greater capacity for oxidizing and unbalancing or degrading intracellular components (Liu et al., 2011a; Chen et al., 2013). Furthermore, Li et al. and Panda et al. proposed that conductive Gr films coated on metallic surfaces siphon electrons from bacterial membranes, which leads to the generation of oxidative stress toxic for the cells (Li et al., 2014; Panda et al., 2018).

The wettability of GMs is another important characteristic impacting on interactions with bacteria. Graphene is generally considered to have a hydrophobic surface, whereas GO is amphiphilic (Hegab et al., 2016). Reduced graphene oxide is significantly more hydrophobic than GO but still has some hydrophilic regions. These differences will affect the affinity of bacteria for Gr, GO, and rGO. The factors determining the wettability of GMs have been extensively studied, and more information can be found in comprehensive review articles (An et al., 2017; Feng and Guo, 2019). Hydrophobicity of microbial surfaces varies from one species to the other and is also influenced by environmental conditions (Krasowska and Sigler, 2014). This suggests that some bacteria may have more affinity toward Gr, whereas others will be more attracted to GO. In the case of BESs, strong bacterial affinity to GMs will lead to better adhesion, facilitate electron transfer, and increase the overall system performance. On the contrary, for antibacterial coatings, GMs with suitable hydrophobicity and surface free energy can impede the attachment of cells with low affinity and prevent biofilm formation (Muthu et al., 2018; Gu et al., 2018; Dubey et al., 2018). For instance, titanium coated with a hydrophobic Gr film exhibiting low surface free energy hindered the adhesion of both Gram-negative and Gram-positive bacterial species (Busscher et al., 1984; Agarwalla et al., 2019).

## Proposed Bactericidal Mechanisms of Graphene

**Figure 2:**
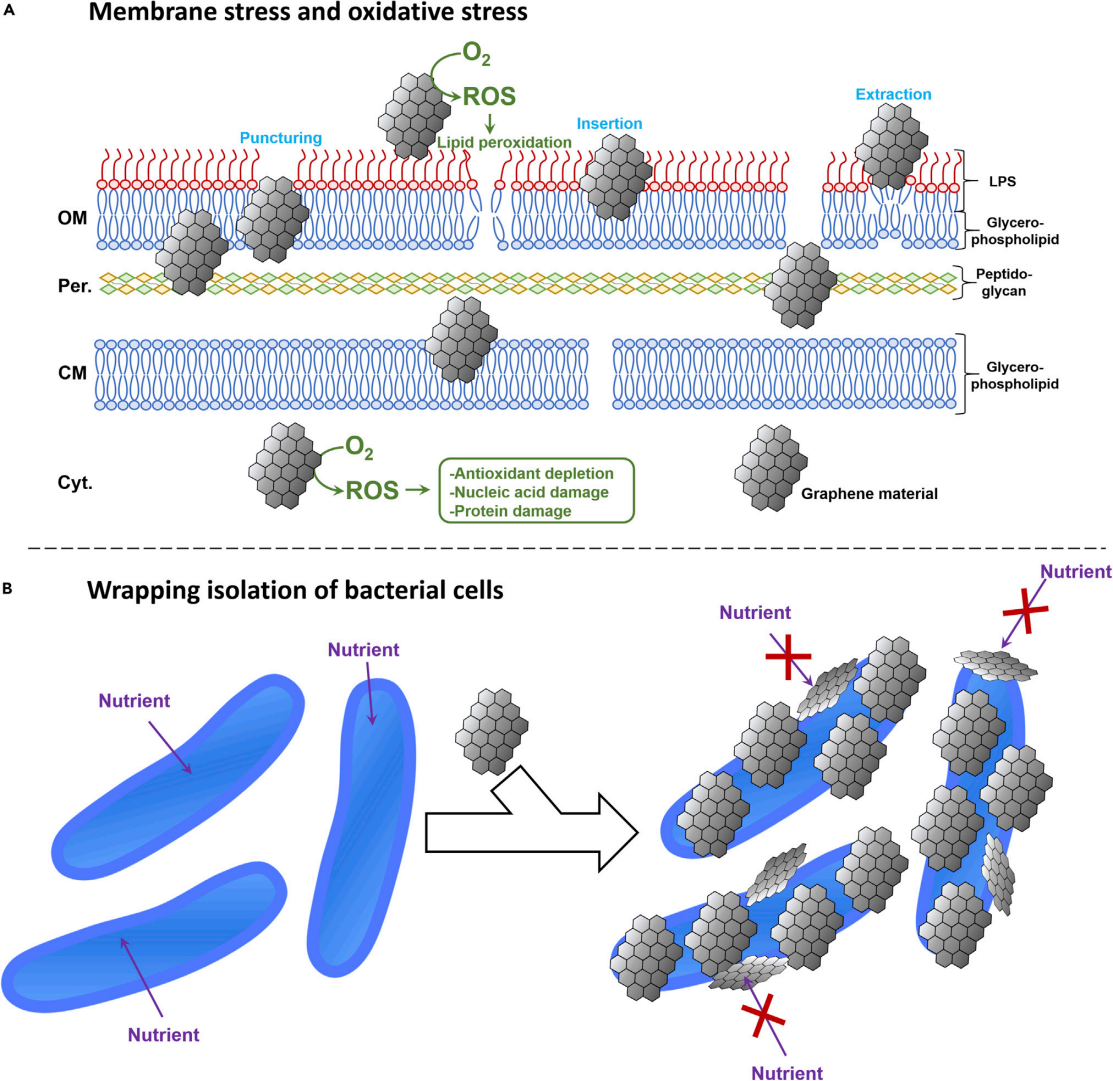
Bactericidal Mechanisms of GMs (A) Membrane and oxidative stress. Membrane stress mechanisms are in blue. Oxidative stress mechanisms are in green. The cell wall structure is from a Gram-negative bacterium. GMs can also damage Gram-positive bacteria via oxidative and membrane stress. (B) Wrapping isolation of bacterial cells.

Besides repulsing bacterial adhesion, GMs with antibacterial properties can also directly damage or starve prokaryotic cells. Three main mechanisms are thought to participate in the bactericidal activity of GMs: membrane stress, oxidative stress, and wrapping isolation (Figure 2) (Hegab et al., 2016; Zou et al., 2016; Shi et al., 2016; Rojas-Andrade et al., 2017). For membrane stress, GMs destroy bacteria by piercing and extracting phospholipids from the membrane(s) safeguarding cell's integrity (Tu et al., 2013). Oxidative stress involves reactive oxygen species (ROS) such as superoxide radical (O_2_^.-^), hydroxyl radical (OH^−^), and hydrogen peroxide (H_2_O_2_) that can be generated by GMs in the presence of bacteria (Perreault et al., 2015a; Zou et al., 2016). Bacterial nucleic acid, membrane lipids, and proteins exposed to oxidative stress will be oxidized and degraded leading to cell destruction (Gurunathan et al., 2012; Perreault et al., 2015b). Lipid peroxidation is a critical phenomenon responsible for the disorganization of cell membrane upon exposure to ROS-generating GMs. ROS react with membrane lipids leading to the formation of lipid peroxides, which in turn oxidize and degrade other membrane components (Krishnamoorthy et al., 2012). Wrapping isolation occurs when bacterial cells are encased in GM sheets and thus separated from their growth medium (Liu et al., 2011a, 2012; Akhavan et al., 2011). This mechanism has been observed with both Gr and GO nanosheets preventing nutrients to pass through the cell membrane and leading to growth inhibition.

## Proposed Bacterial Growth-Enhancement Mechanisms of Graphene

**Figure 3:**
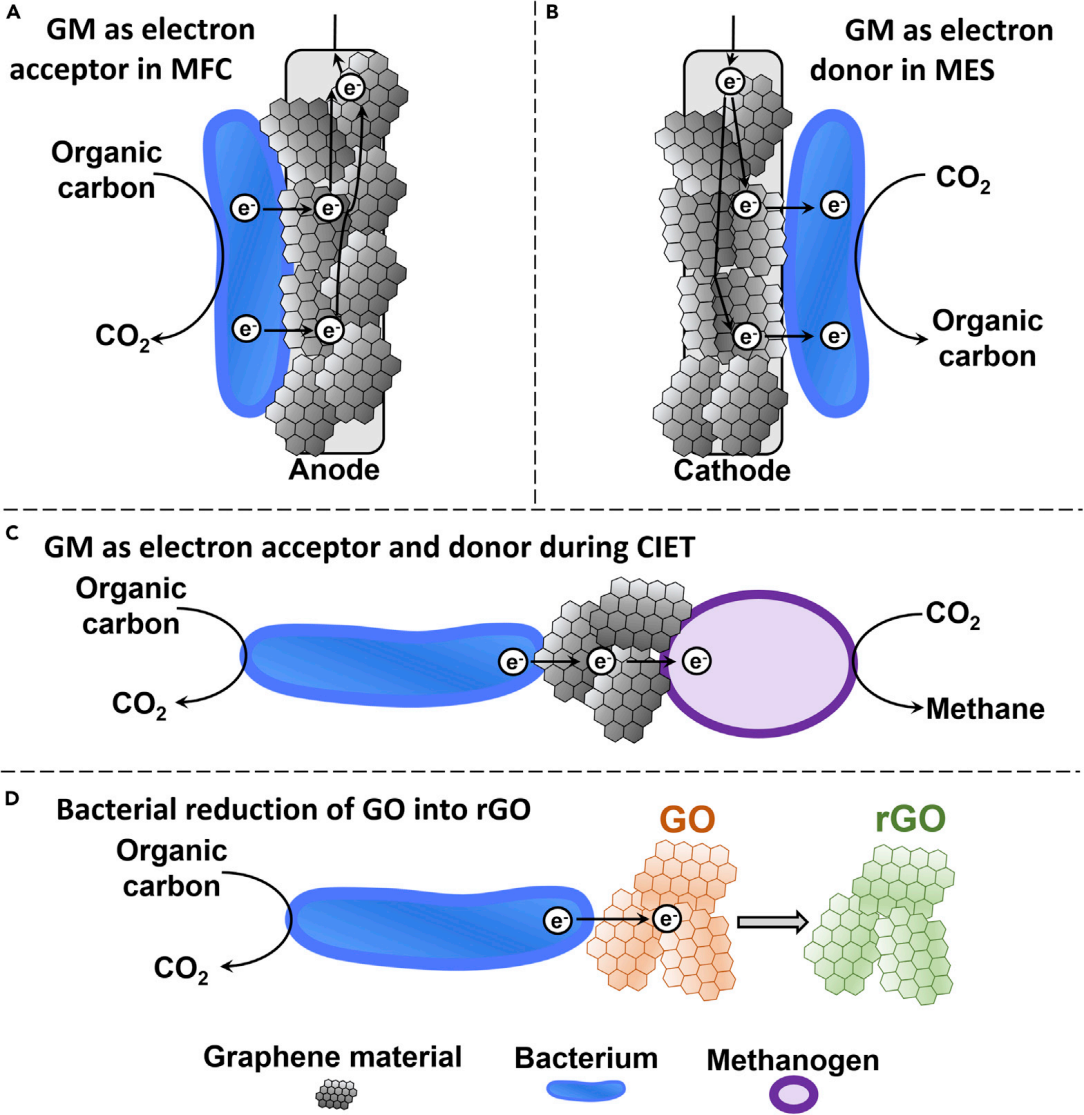
Bacterial Growth-Promoting Mechanisms of GMs Related to Anaerobic Respiration (A) GMs coated on the anode of an MFC serve as the electron acceptor for electroactive bacteria oxidizing organic carbon molecules. (B) GMs coated on the cathode of an MES reactor serves as the electron donor for bacterial CO_2_ reduction. (C) For CIET, GMs serve simultaneously as the electron acceptor for bacteria oxidizing organic carbon molecules and as the electron donor for a second microbe. In this example, a methanogen is receiving electrons from GM to reduce CO_2_ into methane. (D) Bacteria transfer electrons to GO for the synthesis of rGO.

Graphene materials increasing bacterial growth serve as a substrate for cell attachment and proliferation and/or have specific functions in cellular metabolism. For instance, well-cleaned GO enhanced the growth of the model bacterium *Escherichia coli* by promoting cell attachment and biofilm formation (Ruiz et al., 2011). In BESs, GM coatings on electrodes have a specific role in the anaerobic metabolism of electroactive bacteria (Figure 3). Graphene material coatings on the anode of MFCs serve as the electron acceptor for microbes generating current by oxidizing organic carbon molecules (ElMekawy et al., 2017). Graphene oxide can also be used as the electron acceptor by bacteria for the synthesis of rGO (Salas et al., 2010). In microbial electrosynthesis (MES) reactors, GM coatings on the cathode act as the electron donor for the conversion of electrical energy and the greenhouse gas CO_2_ into valuable chemicals (Tremblay et al., 2020). In anaerobic digesters, GMs can be employed simultaneously as the electron donor and the electron acceptor by different bacterial species and thus function as electron bridges for interspecies electron transfer (IET) (Tian et al., 2017).

## Factors and Circumstances Determining the Positive or Negative Impact of Graphene on Bacteria

Multiple scientific publications are reporting that GMs have antibacterial effects. In parallel, a large number of studies are indicating that GMs can promote bacterial growth and/or biofilm formation (Hegab et al., 2016; Perreault et al., 2015a; Ruiz et al., 2011; ElMekawy et al., 2017; Tremblay et al., 2020). This suggests that antibacterial mechanisms associated with GMs are active only under specific circumstances and probably required GM with certain physicochemical properties. Likewise, specific conditions must be met for GMs to have a beneficial impact on bacterial growth. The following sections discuss the different factors and circumstances that may affect fundamentally GM-bacteria interactions including the physicochemical characteristics of GM, the combination of GM with other material into composites, the GM dose, the GM synthesis process, the presence of O_2_ in the environment, the bacterial species exposed to GM, the bacterial culture density, and the general growth conditions (Table 1).

**Table 1:** Factors Determining the Impact of GMs on Bacterial Growth and Metabolism and Potential Applications

Factors with a Positive or Negative Impact
Size, morphology, SSA, roughness, hydrophobicity/hydrophilicity, GM composite, bacterial species, bacterial growth stage, culture and environmental conditions, low or high GM dose
Factors with a positive impact only Anaerobic growth condition
Applications based on positive interactions between bacteria and GMs MFC, MES, CIET, and methane production by anaerobic digestion, bacterial reduction of GO into rGO
Factors with a negative impact only Toxic carryover from synthesis, presence of O_2_
Applications based on negative interactions between bacteria and GMs Environmental Packaging, PPE fabrics, antifouling membranes for water treatment and metal recovery, photocatalysts for water disinfection, hydrogel filters, aggregates for water treatment, coatings to prevent MIC Medical Drug delivery systems, tissue engineering scaffolds, wound dressings, medical equipment, and implantable device coatings

GM, graphene material; SSA, specific surface area; MFC, microbial fuel cell; MES, microbial synthesis; CIET, conductive-material-mediated interspecies electron transfer; GO, graphene oxide; rGO, reduced graphene oxide; PPE, personal protective equipment; MIC, microbially influenced corrosion.

### Graphene Size, Morphology, Surface Area, and Roughness

Multiple physicochemical characteristics of GM have an impact on its interactions with bacteria and its toxicity level (Hegab et al., 2016; Perreault et al., 2015a). For surface coating, GMs with a smaller sheet size are associated with greater toxicity because of their higher capacity for mechanical cell membrane disruption and ROS generation (Dallavalle et al., 2015; Perreault et al., 2015b). For instance, the antibacterial activity of GO nanosheets of 0.01 μm^2^ coated on a filter was four times higher compared with that of GO nanosheets of 0.65 μm^2^ (Perreault et al., 2015b). The sharp edges of GM nanosheets are particularly important for membrane stress as they enable efficient penetration of the phospholipid bilayers (Akhavan and Ghaderi, 2010; Li et al., 2013). Interestingly, in suspension, GO nanosheets with higher lateral size can inhibit bacterial growth more efficiently by cell wrapping, but only in a temporary and reversible manner (Liu et al., 2012; Perreault et al., 2015b).

Specific surface area and roughness are other attributes varying from one GM to the other that may affect microbial interactions and biofilm formation. For instance, the larger SSA of GM compared with other materials probably means that more sites are available for microbial interaction and adhesion. Rough surfaces with frequent irregularities have higher suitability for microbial colonization than smooth surfaces (Terada et al., 2005). Thus, higher SSA and roughness augments the number of bacterial contacts, which may lead to either increased antibacterial effects or biofilm formation depending on the context.

### Graphene Composites for Antibacterial Applications

An important circumstance determining the impact of GM on bacteria is either if it is employed in a pristine form or combined with another material into composite.

Many GMs-based composites with metals, natural or synthetic polymers, sand, antibiotics, enzymes, hydroxyapatite, and hydrogels have been developed to accentuate antibacterial properties as well as to improve other critical characteristics for different applications such as thermal stability, porosity, and mechanical resistance (Poornima Parvathi et al., 2015; Lim et al., 2012; Liu et al., 2014; Janković et al., 2015; Duan et al., 2015b; Rojas-Andrade et al., 2017; Mohammed et al., 2020). Three main types of GM composites have been fabricated: dispersion, film, and hydrogel. Dispersions are developed to kill or inhibit bacteria present in a solvent. Films are usually employed for antifouling applications. GMs-functionalized hydrogels are mainly fabricated for water purification or wound healing (Wang et al., 2013a; Shen et al., 2018; Fan et al., 2014).

Antibacterial GM composites described until now often comprise a metal (e.g., Ag, Cu, stainless steel) a metal oxide (e.g., Fe_3_O_4_, TiO_2_, ZnO), or a metal sulfide (e.g., CdS) (Hegab et al., 2016). Like GMs, metallic compounds generate ROS leading to oxidative stress highly damaging for living cells (Yao et al., 2020; Zou et al., 2016; Rojas-Andrade et al., 2017). Combining GM with metals probably accentuates ROS-generation capacity in a synergistic manner leading to a more pronounced antibacterial effect. For instance, a composite combining rGO with ROS-generating Ag nanoparticles was fabricated by Xu et al. in a seminal work (Xu et al., 2011). The Ag@rGO exhibited an antibacterial effect against *E. coli* that was higher than pure Ag nanoparticles and comparable with the antibiotic ampicillin. In the case of GMs composites with metal oxides or metal sulfides, they are mainly being developed as photocatalytic materials to drive the generation of cytotoxic ROS by visible light (Deng et al., 2016; Akhavan and Ghaderi, 2009).

### Graphene Composites for Bioelectrochemical Applications

Biocompatible electrodes made of GM composites have been developed for MFC and MES reactors (ElMekawy et al., 2017; Tremblay et al., 2020). A common approach reported in the literature is to fabricate an electrode made of a porous or mesh-like conductive material with a large SSA coated with GMs to maximize electron transfer between the electrodes and the microbes (Zhang et al., 2011b; Song et al., 2018b; Aryal et al., 2019). For these applications, GMs appear to be highly biocompatible and have beneficial interactions with the bacterial catalyst. In the case of MFC, composite anodes have been made with GM combined with metals, metal oxides, metal carbides, other carbonaceous materials, natural and synthetic polymers, zeolite, vitamins, or ionic liquid (Tremblay et al., 2020; Paul et al., 2018; Yu et al., 2018; Li et al., 2020; Islam et al., 2020; Ma et al., 2020; Zou et al., 2019). Besides higher SSA and biocompatibility, GM composites have improved electrical conductivity as well as greater capacity for bacterial adhesion and extracellular electron transfer (EET). Graphene material composite electrodes have also been developed to serve as cathode for CO_2_ reduction by MES. This includes pristine or modified GMs coated on metallic foam or carbonaceous substrate (Aryal et al., 2017a, 2019). Coating the anode of an MFC or the cathode of an MES reactor with GMs usually leads to the formation of denser biofilms where bacterial cells attached themselves readily and have a greater number of direct physical contact with the surface of the electrode, thus augmenting the capacity for electron transfer at the microbe-electrode interface essential for electrical current generation or consumption.

**Figure 4:**
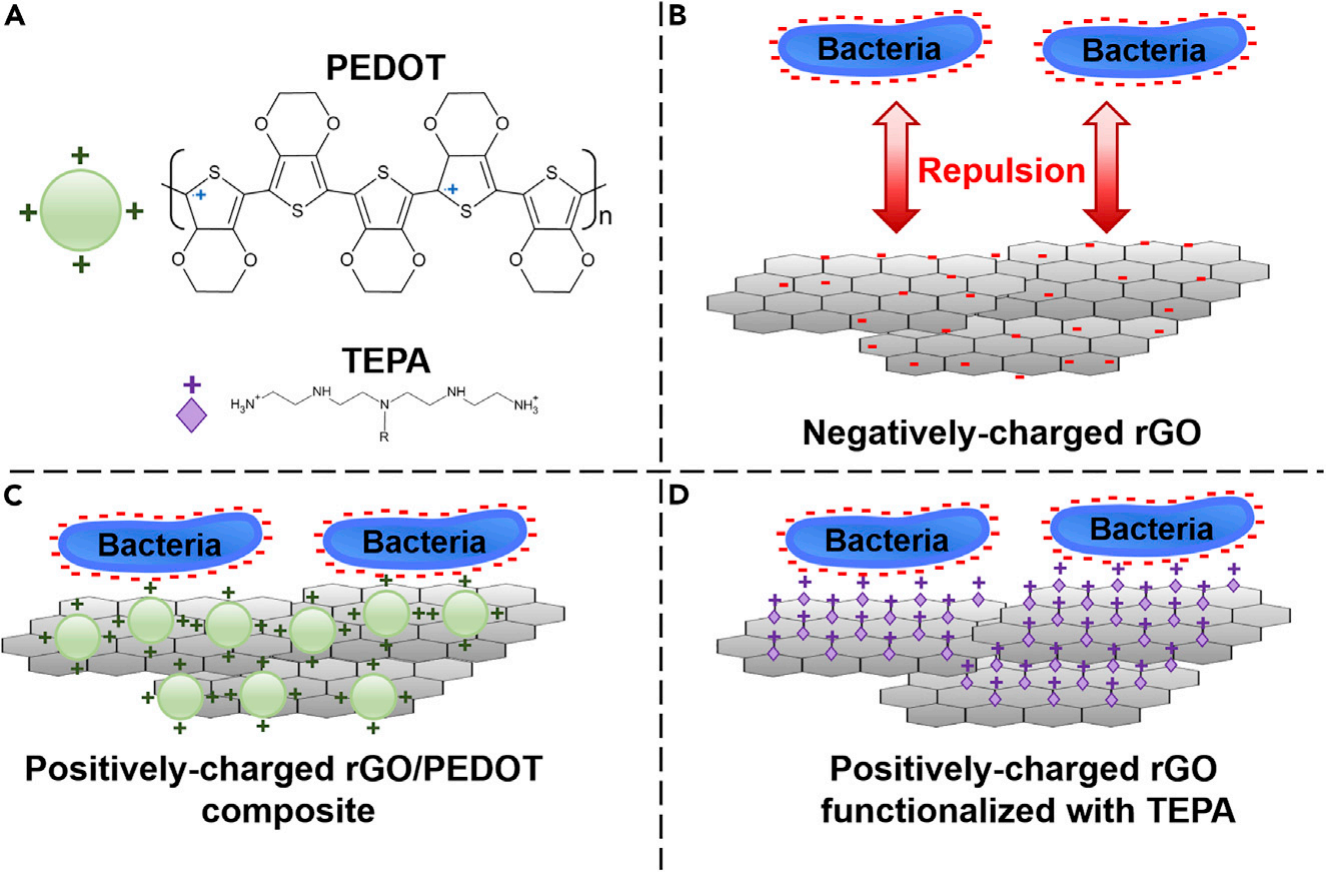
Graphene Materials and Electrostatic Interactions with Bacteria (A) Structure of positively charged poly(3,4-ethylenedioxythiophene) (PEDOT) and tetraethylenepentamine (TEPA). (B) Electrostatic repulsion between negatively charged bacteria and rGO. (C) Attachment of bacteria (*E. coli*) on rGO coated with PEDOT in an MFC (Wang et al., 2013b). (D) Attachment of bacteria (*S. ovata*) on rGO functionalized with TEPA in an MES reactor (Chen et al., 2016b).

One important challenge when trying to develop BES reactors relying on biofilm formation on GO or rGO surfaces is that the overall negative charge of these materials leads to the repulsion of bacteria, which also have a net negative surface charge (Figure 4) (Zhang et al., 2013; ElMekawy et al., 2017; Hegab et al., 2016). A solution to this issue is to change the charge of the GM-containing electrodes by adding another component that is positively charged. For instance, an *E. coli*-driven MFC with an anode made of rGO coated with poly(3,4-ethylenedioxythiophene) (PEDOT), a conducting polymer with a positively charged backbone, had an improved biofilm formation (Wang et al., 2013b). Consequently, the rGO/PEDOT-coated anode exhibited a maximum power density of 873 mW m^−2^, which was 15 times higher than a control uncoated anode. For MES cathode, this problem has been resolved by employing rGO functionalized with positively charged tetraethylene pentamine (TEPA) (Chen et al., 2016b). MES with rGO-TEPA exhibited higher biofilm formation by the negatively charged acetogen *Sporomusa ovata*. This led to a rate of acetate production from CO_2_ and electricity of 2.6 g L^−1^ h^−1^ per m^2^ of electrode that was 11.8 times higher with the cathode coated with rGO-TEPA compared with the uncoated cathode.

### Graphene Materials Dose

Multiple studies have reported that the toxicity of GMs is dose dependent (Table 1). For instance, Gurunathan et al. showed that both GO and rGO at concentrations below 25 mg L^−1^ had no impact on the viability of the Gram-negative opportunistic pathogen *Pseudomonas aeruginosa* (Gurunathan et al., 2012). A reduction of viability greater than 50% was observed when the concentration of GO or rGO was increased to 100 mg L^−1^. Similar observations were made for the model bacterium *E. coli* with no cell viability loss in the presence of 25 mg L^−1^ GO or rGO (Gurunathan et al., 2013a). Increasing GMs concentration to 100 mg L^−1^ resulted in viability losses of *E. coli* culture close or below 50%. Song et al. measured biofilm formation by *E. coli* and by the Gram-positive model bacterium *Bacillus subtilis* in the presence of different concentrations of GO (Song et al., 2018a). Interestingly, biofilm formation by both bacteria was significantly increased with 10 mg L^−1^ GO. The statistically significant reduction of biofilm formation was observed only when GO concentration was above 80 and 160 mg L^−1^ for *E. coli* and *B. subtilis*, respectively. These results suggest that the effect of GMs can be alternated between promoting or inhibiting bacterial growth by simply modulating the dose to which cells are exposed.

### Toxic Compounds Carried over from Synthesis Processes

For GO, Ruiz et al. argued that it had no intrinsic bacteriostatic or antibacterial properties and that bacterial growth is accelerated in its presence (Ruiz et al., 2011). Based on these observations, the authors suggested that the negative impact of GMs on bacteria observed in multiple studies is, in fact, caused by toxic impurities carried over during the synthesis process. Ruiz et al. did multiple rounds of extensive dialysis and dilution to clean GO and establish its biocompatibility, whereas other groups may not have performed sufficient washing after synthesis leaving impurities that can disrupt bacterial cell membranes and impede multiple cell functions. Graphene oxide and rGO are usually synthesized from graphite by modified versions of the Hummers' method followed by a reduction step in the case of rGO (Hummers and Offeman, 1958; Sun et al., 2015; Yu et al., 2016; Zhu et al., 2013; Song et al., 2018a; Hu et al., 2010; Liu et al., 2011a). This process involves multiple chemicals that are highly deleterious for living cells and that must be extensively removed prior to microbial viability experiments such as sulfuric acid, hydrogen peroxide, potassium permanganate, and hydrazine. With the accumulation of reports demonstrating the bacterial growth-promoting activity of GMs, the possibility that antimicrobial effects previously attributed to GMs are in fact related to carry-over of toxic chemicals from synthesis must be considered with care. Another aspect to examine closely is that the synthesis processes for GMs are not standardized properly and many companies or laboratories worldwide are producing poor-quality GMs with a high level of contaminants (e.g., metals) or are even misrepresenting their product and fabricating entirely different compounds such as graphite platelets (Kauling et al., 2018). Thus, it is important to abide by strict production and characterization criteria for GMs when investigating their effects on bacterial cells.

### Aerobic Versus Anaerobic Environment

Major mechanisms responsible for the toxicity of GMs on bacteria are linked to oxidative stress related to ROS (Figure 2) (Yao et al., 2020; Zou et al., 2016). Oxidative stress mediated by GMs required the presence of molecular O_2_ (Krishnamoorthy et al., 2012; Gurunathan et al., 2012, 2013a; Liu et al., 2011a). In the presence of bacteria, GMs absorb O_2_ from the environment on its edges and defects, which will then be reduced by bacterial enzymes such as glutathione-dependent enzymes (Perreault et al., 2015a; Zou et al., 2016; Liu et al., 2011b). Subsequently, ROS toxic for bacterial cells are released from the surface of GMs. This is accompanied by the depletion of cellular antioxidants such as glutathione, which increases the vulnerability of bacterial cells toward oxidative stress (Perreault et al., 2015b; Liu et al., 2011a).

The vast majority of experiments investigating the toxicity of GMs for bacteria has been done under aerobic conditions. On the contrary, BESs with a GM-comprising bioanode or biocathode are usually maintained under strict anaerobic growth conditions with constant gas flushing to remove O_2_. In the case of BES with a bioanode such as MFC, anaerobic conditions are established in the anode chamber because the bacteria driving electrical current generation are often strict anaerobes (Franks and Nevin, 2010). Facultative anaerobes are also employed as microbial catalysts for MFC (Cao et al., 2019; Logan et al., 2019). Besides its high toxicity for certain electroactive bacteria, O_2_ is removed from the anodic chamber because it can be used as a terminal electron acceptor (TEA) preferentially to the anode and thus inhibits electrical current generation. For BESs with biocathode such as MES, the pure culture or mixed community acquiring electrons from the electrode are mostly strict anaerobes (Tremblay and Zhang, 2015; Nevin et al., 2010; Kracke et al., 2018). Furthermore, O_2_ contamination in the cathodic chamber would interfere with the electron transfer between the cathode and microbes since a large part of the electrode's reducing power would be hijacked for O_2_ reduction. Consequently, the absence of O_2_ in BESs may be a major reason why GMs-comprising bioelectrodes promote bacterial proliferation instead of preventing it.

### Bacterial Species, Growth Stage, Culture, and Environmental Conditions

Other important factors influencing the interaction between GMs and bacteria include growth medium composition, pH, salinity, bacterial species exposed to GMs, and growth stage (Table 1) (ElMekawy et al., 2017). For instance, the cell wall structure differs between bacterial species, which has an impact on the cell capacity to tolerate stresses such as ROS-related lipid peroxidation and membrane disruption by GMs with sharp edges (Perreault et al., 2015b; Krishnamoorthy et al., 2012; Gurunathan et al., 2012; Tu et al., 2013). This is illustrated by the observation that the Gram-negative bacterium *E. coli* with an outer membrane and a cytoplasmic membrane was more resistant to GO nanowall edges than the Gram-positive bacterium *Staphylococcus aureus*, which has only a cytoplasmic membrane with a thick peptidoglycan layer (Akhavan and Ghaderi, 2010). Additionally, bacterial species have a net surface negative charge with variable electrostatic strength affected by medium composition, pH, growth stage, and so on (Hegab et al., 2016). The negative charge level at the cell's surface will influence bacterial adhesion to pristine or modified GMs and have an impact on the nature of the interaction between GMs and the microbe. Besides its effect on the physicochemical properties of bacteria, the growth stage also determines cellular density. Since GMs toxicity is dose dependent, it is likely that adding the same GMs concentration in a bacterial culture at an early growth stage with lower cell density could be toxic but non-toxic at a later growth stage in the presence of more cells.

Besides impacting bacterial metabolism, culture and environmental conditions also affect GM stability and conformation (Palmieri et al., 2017a). For instance, Palmieri et al. (2017) showed that GO can be destabilized and changes conformation via salt-dependent DLVO-like aggregation (Palmieri et al., 2017b). In fact, GO at a low concentration in ultrapure water, phosphate-buffered saline (PBS), or different salt solutions is stable and its edges disrupt bacterial cell membranes. When the concentration of GO is increased, it loses its antibacterial activity in all the tested solutions excepted ultrapure water. In these environmental conditions, high-concentration GO is destabilized and forms small aggregates where its bactericidal edges are now hidden. At an even higher concentration in salt solutions, GO recovers its capacity to kill bacteria because GO aggregates become larger and can now wrap cells completely, thus hindering bacterial growth. These observations illustrated well the complex assembly of factors such as environmental conditions and material dose that affects how bacterial cells will react to GMs.

## Applications for Positive Bacterial Interactions with Graphene

Several applications exploiting the positive effect of GMs on bacteria are currently being investigated by different research groups (Table 1). Graphene materials have been extensively employed for the fabrication of better electrodes with high conductivity and better SSA maximizing the quantity of physical contacts with bacterial cells for both power generation by MFC and the conversion by MES of CO_2_ and electrical energy into valuable multicarbon compounds (ElMekawy et al., 2017; Tremblay et al., 2020). Graphene materials, like other carbonaceous materials, can also enable conductive-material-mediated interspecies electron transfer (CIET) and enhance the conversion of organic carbon compounds from wastes into methane by anaerobic digestion (Dang et al., 2016; Igarashi et al., 2020). Furthermore, bacteria can be integrated into the synthesis process of GMs by catalyzing the reduction of GO into rGO (Hegab et al., 2016).

### MFC with Graphene-Comprising Anode

In recent years, GMs have been included in the design of multiple high-performance anodes for MFC applications (ElMekawy et al., 2017; Yuan and He, 2015; Huang et al., 2011). MFC design can vary significantly between reactors, but the core of the system is the anodic chamber where microbes oxidize organic carbon molecules and transfer the resulting electrons to an anode (Figure 3A). With this technology, microbes generate electricity by extracting chemical energy from different types of wastewater (Liu et al., 2017; Pant et al., 2010).

Extracellular electron transfer between the microbial catalyst and the anode is a critical step in the MFC process and is often a limiting one because of low efficiency. Extracellular electron transfer can be direct or indirect. Direct EET requires physical contacts between microbial cells and/or biofilm with the anode surface and involves components of the cell wall such as *c*-type cytochromes and type-IV pili (Zhang et al., 2019; Reguera, 2018; Li et al., 2018a; Malvankar et al., 2011; Kumar et al., 2016; Cotts et al., 2020). Indirect EET mostly relies on soluble mediators carrying electrons from the microbes to the anode (Saratale et al., 2017; Zhou et al., 2013; Kotloski and Gralnick, 2013). Extracellular electron transfer efficiency will depend on multiple parameters including the anode material and configuration (ElMekawy et al., 2017). Optimal anode surfaces for microbial EET must exhibit essential characteristics associated with GMs such as low cost, high electrical conductivity, biocompatibility, flexibility, and porosity (Li et al., 2017a).

Historically, anodes for MFC have often been fabricated with carbonaceous materials such as graphite and carbon cloth (CC). These materials are conductive and they are stable when compared with metals susceptible to corrosion (Li et al., 2017a; Picot et al., 2011; Song et al., 2012; Chaudhuri and Lovley, 2003; Liu and Logan, 2004; Logan et al., 2007; Wang et al., 2009). Graphene materials present additional benefits for the fabrication of MFC anodes such as outstanding SSA and better electrical conductivity and, thus, have been shown to improve significantly biofilm formation, EET transfer rate at the bacteria-anode interface, and power density (Sonawane et al., 2017; Liu et al., 2017; ElMekawy et al., 2017).

Anode surfaces for MFC have been modified with GM only, GM with conductive polymer composite, or GM with metal composite (Tremblay et al., 2020). Examples of MFC anode leading to high power density (per m^2^ of electrode surface) with GM-only surface includes stainless steel mesh (SSM) coated with rGO (Zhang et al., 2011b). In this case, the presence of rGO on the surface of SSM increased maximum power density to 2.67 W m^−2^, which was 18 times better than the uncoated SSM. The authors attributed the higher power generation to enhanced adhesion of *E. coli* to rGO and more efficient EET from the microbes to the anode. In another study, an MFC anode was made of CC coated with rGO sheets crumpled by capillary compression (Luo et al., 2011). This alteration of rGO spatial configuration aimed at increasing the SSA available for EET with the microbial catalyst and improved maximum power density up to 2.4 W m^−2^. These studies by Zhang et al. and Luo et al. are some of the first reports on the advantages of GM-based anodes for power generation by MFC (Zhang et al., 2011b; Luo et al., 2011).

The fabrication of MFC anodes coated with GM can also be done with non-conductive GO (Yong et al., 2014; Yuan et al., 2012; Lin et al., 2018). With this approach, microbes reduce GO into conductive rGO and form a three-dimensional rGO-biofilm hybrid on the surface of CC (Figure 3D). For instance, Yuan et al. injected GO to an anodic chamber already colonized with microbes from anaerobic sludges (Yuan et al., 2012). GO was then microbially reduced into rGO, which formed a network on the surface of a CC electrode leading to a maximum power density of 1.91 W m^−2^.

There are multiple examples of MFCs with enhanced power density equipped with anodes coated with GM-polymer composites (Tremblay et al., 2020). This includes GM combined with polydopamine (PDA), polytetrafluoroethylene (PTFE), agarose, polyaniline (PANI), polypyrrole (PPy), polyurethane (PU), the polyelectrolyte poly(allylamine hydrochloride) (PAH), Nafion, and (Poly N-Isopropylacrylamide) (PNIPAM) hydrogel (Li et al., 2020; Kirubaharan et al., 2015; Hou et al., 2013; Gnana kumar et al., 2014; Zhu et al., 2014; Li et al., 2018b; Xie et al., 2012; Hou et al., 2014; Yang et al., 2015; Zhao et al., 2013a; Yong et al., 2012; Kumar et al., 2014). These composites generally improve the porosity of the electrode, SSA available for microbial interactions, electrical conductivity, and adhesiveness for biofilm formation and facilitate EET from microbes. More recently, an MFC equipped with a carbon paper (CP) anode coated with a composite made of rGO and the polyelectrolyte poly(diallyldimethylammonium chloride) (PDDA) prepared in a Nafion solution exhibited a superior maximum power density of 5.03 W m^−2^ (Ma et al., 2020). The polyelectrolyte PDDA had a synergistic positive effect with rGO on power density by enhancing electron attraction by the anode, thus leading to more efficient EET. The polyelectrolyte also increased SSA and surface biocompatibility resulting in the quick formation of a stable electroactive biofilm.

Graphene materials with noble metals, metal oxides, or metal carbides are other types of composite frequently investigated for the coating of performant MFC anodes (Zou et al., 2019; Mehdinia et al., 2014; Zhao et al., 2014, 2015a, 2015b; Song et al., 2016; Fu et al., 2020). For instance, a *Shewanella oneidensis*-driven MFC equipped with a three-dimensional Gr aerogel anode decorated with Pt nanoparticles had a maximum power density of 1.46 W m^−2^ (Zhao et al., 2015b). The fabrication of GM-based composite for MFC anode with metal oxides instead of noble metals could be more viable because of lower cost. This is illustrated by the good performance with power densities above 1 W m^−2^ of MFCs equipped with anodes coated with GM-SnO_2_ or GM-TiO_2_ composites (Mehdinia et al., 2014; Zhao et al., 2014). Besides being more affordable, metal oxide semiconductors like SnO_2_ or TiO_2_ are chemically stable and biocompatible and have a high SSA. A third option for GM-metal composites in the fabrication of MFC anodes is metal carbides, which have shown good electrocatalytic activities in BESs (Rosenbaum et al., 2006, 2007). Recently, a CC anode coated with a composite made mainly of Mo_2_C and Gr was tested in an MFC colonized by the electroactive *bacterium Shewanella putrefaciens* (Zou et al., 2019). Maximum power density with the composite was 1.70 W m^−2^, which was 2-fold higher than Gr only, thus demonstrating the advantage of combining GMs with other high-performance conductive materials for MFC applications.

Anode surfaces for MFC have also been fabricated with GM blended with other types of material such as CNT, zeolite, ionic liquid, and vitamin B_2_ (Song et al., 2016; Kumar et al., 2014; Paul et al., 2018; Yu et al., 2018; Zhao et al., 2013b). In all cases, GMs establish a positive synergistic relationship with the other compound included in the composite leading to significant improvements of the MFC performance.

### MES with Graphene Cathode

In MES reactors, microbes are colonizing the cathodic chamber where they reduce CO_2_ into valuable carbon-based chemicals with electrons derived from a cathode (Figure 3B) (Rabaey and Rozendal, 2010; Tremblay et al., 2017; Lovley and Nevin, 2011; Tremblay and Zhang, 2015; Zhang and Tremblay, 2019; Nevin et al., 2010; Prévoteau et al., 2020; Aryal et al., 2017a; Ammam et al., 2016). As for MFC, EET between the electrode and microbes is the core of the MES process and its rate and efficiency will be impacted by multiple factors including electrode material. Graphene materials are highly promising for the fabrication of MES cathodes because their physicochemical characteristics are suitable to promote fast microbial-driven EET. Cathodes made of freestanding rGO paper, of nickel foam coated with Gr, of CC coated with rGO-TEPA, and of rGO on carbon felt (CF) outperformed comparable cathodes without GMs for the MES of acetate from CO_2_ (Song et al., 2017, 2018b; Aryal et al., 2017b; Chen et al., 2016b).

**Figure 5:**
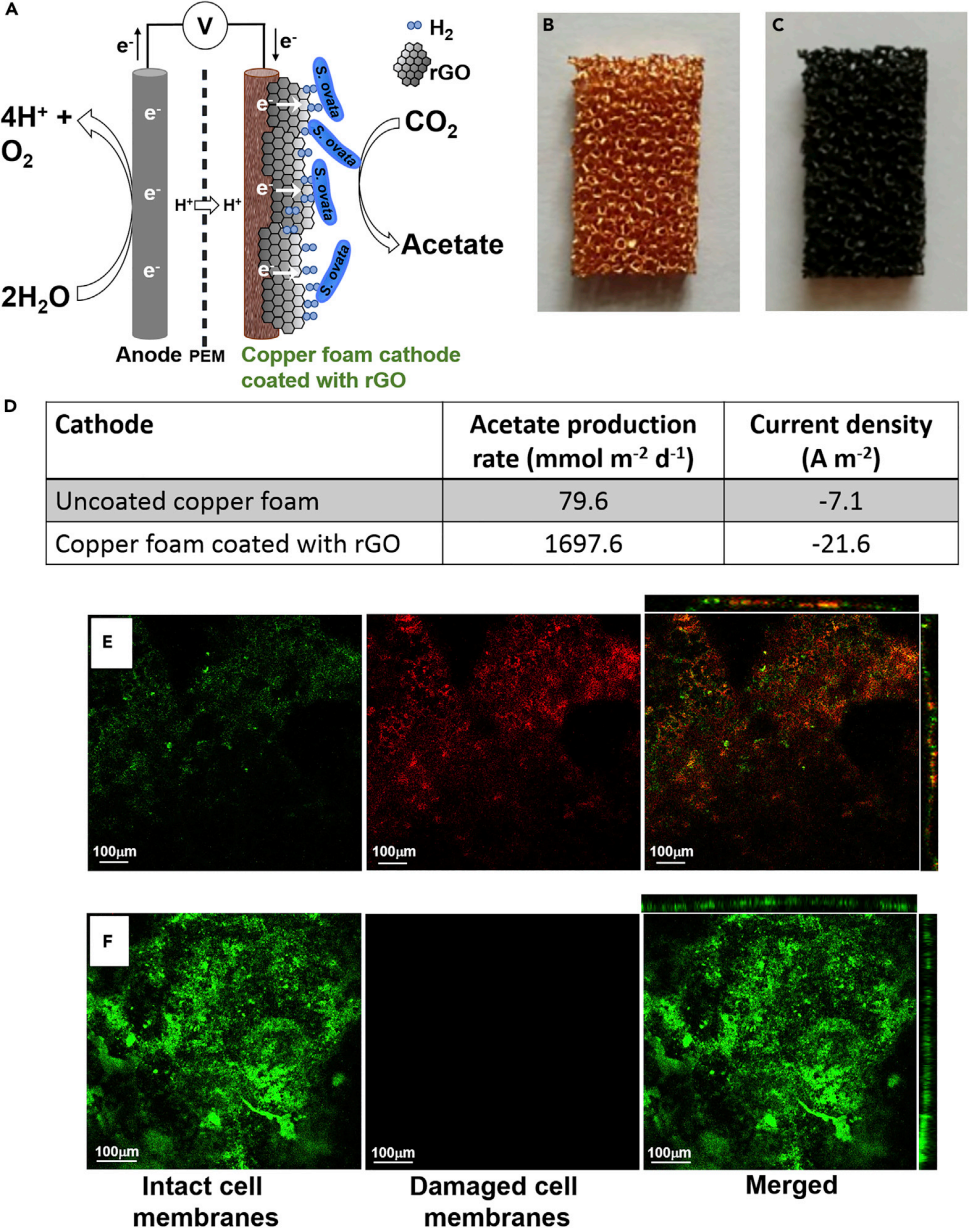
Reduced Graphene Oxide Coating Protects Bacterial Cells from Toxic Copper and Promotes Biofilm Formation in MES Reactor (A–C) (A) Scheme of an *S. ovata*-driven MES reactor with a copper foam cathode coated with rGO. PEM, proton-exchange membrane. (B) Uncoated copper foam and (C) copper foam coated with rGO. (D) Acetate production rate and current density during MES with either cathode. (E and F) (E) Confocal laser scanning microscopy images with live/dead staining after 10 days of MES operation with (E) uncoated copper foam cathode and (F) copper foam cathode coated with rGO. For (E) and (F), cells stained in green have an intact membrane, whereas cells stained in red have a damaged membrane. Figure adapted from Aryal et al. (2019) with permission from Elsevier.

A clear demonstration of the bacterial growth-promoting activity of rGO in MES reactors was achieved with a copper foam cathode coated with rGO with the Gram-negative acetogen *S. ovata* as the microbial catalyst reducing CO_2_ (Figure 5) (Aryal et al., 2019). In this system, confocal laser scanning microscopy with live/dead stain showed that, as expected, an uncoated copper foam cathode inhibited bacterial growth due mainly to the release of toxic Cu^2+^. Copper is commonly used for electrochemical applications because of its very high electrical conductivity and relatively low price. However, it has well-characterized antimicrobial properties limiting its utilization as electrode material for MES (Lemire et al., 2013; Grass et al., 2011). When the copper foam was coated with rGO, metabolically active bacterial cells proliferated and a healthy biofilm was formed on the cathode. MES with the copper foam-rGO cathode had a superior performance with an acetate production rate of 1,697 mmol per day per m^2^ of electrode, an average current density of −21.6 A m^−2^, and a coulombic efficiency of 76.4%. Besides higher MES performance, these results also demonstrated that GMs can be employed as a conductive and biocompatible buffer between a biofilm and another material with outstanding electrochemical properties, but with an antibacterial surface.

### Graphene as an Accelerating Agent for IET

Interspecies electron transfer is a process where a microbial cell serves as an electron acceptor for a second microbial cell (Shrestha and Rotaru, 2014). This phenomenon has critical importance in multiple environments for biogeochemical cycles and the complete degradation of organic matter into methane (McInerney et al., 2009). Interspecies electron transfer can be indirect via soluble redox mediators. It can also be direct (DIET) with physical contact between two microbial cells enabling electron transfer via cellular components such as *c*-type cytochromes and conductive pili (Lovley, 2017; Liu et al., 2018; Yin and Wu, 2019; Walker et al., 2020; Summers et al., 2010; Rotaru et al., 2014).

Conductive materials including biochar, granular activated carbon, and Fe oxides can also serve as bridges for IET (Shrestha and Rotaru, 2014; Xu et al., 2019a; Park et al., 2018a, 2018b; Wang et al., 2018; Li et al., 2017b; Zhang et al., 2017b; Kato et al., 2012; Jiang et al., 2013). Interestingly, CIET is exploitable for industrial purposes. Adding conductive materials to biogas-producing anaerobic digesters accelerated the conversion of organic matter into methane probably by facilitating IET between bacteria degrading organic carbon molecules and methanogens (Cruz Viggi et al., 2014; Yamada et al., 2015; Park et al., 2018a; Dang et al., 2016; Zhao et al., 2018b).

Graphene materials are some of the conductive materials that increased methane production when added to an anaerobic digester (Tian et al., 2017; Lin et al., 2017). A recent study by Igarashi et al. demonstrated rGO-mediated CIET between the electroactive bacterium *Geobacter metallireducens* and the methanogen *Methanosarcina barkerii* (Figure 3C) (Igarashi et al., 2020). In this experiment, *G. metallireducens* oxidized ethanol and reduced solid GO to rGO. Subsequently, rGO promoted electron transfer to *M. barkeri* for methane production from CO_2_. The same report also showed that adjusting the hydrophilicity level of rGO has a significant impact on bacterial interactions. *G. metallireducens* adhered faster to rGO exhibiting higher hydrophilicity, which led to more efficient CIET with the methanogen. These studies highlight the industrial potential of biocompatible GMs as conductive materials for the production of bioenergy under the form of methane.

### Bacterial Reduction of GO for the Synthesis of rGO

Another example of application relying on a positive interaction between bacteria and GM is the biological reduction of GO into rGO. Several species capable of EET to solid electron acceptors can perform the reduction of GO into rGO (Figure 3D). This includes *Shewanella*, *Desulfuromonas*, and *Geobacter* species and probably involves the participation of bacterial *c*-type cytochromes as well as soluble electron shuttles (Yoshida et al., 2016a, 2016b; Jiao et al., 2011; Salas et al., 2010; Wang et al., 2011; Lu et al., 2020). *E. coli* and *P. aeruginosa* are other bacteria shown to catalyze the reduction of GO into rGO (Gurunathan et al., 2013b, 2013c; Akhavan and Ghaderi, 2012; Zhao et al., 2018a). Besides being exploited for the design of MFC's bioanode or CIET, the capacity of electroactive bacteria to reduce GO served for the development of oxygen evolution reaction (OER) electrocatalysts (Kalathil et al., 2019). In the system of Kalathil et al., *G. sulfurreducens* reduced GO and formed a simple and durable rGO-based biohybrid electrocatalyst doped with active elements such as Fe and Cu. The bacterial reduction of GO is a promising method that can be integrated into the fabrication of GMs for diverse applications. Its lower cost and non-toxicity represent clear advantages over chemical routes of GO reduction (Raveendran et al., 2013; Lehner et al., 2019).

## Applications for Negative Bacterial Interactions with Graphene

Graphene materials designed to inhibit bacterial growth are being explored for multiple technological applications (Table 1). For instance, GMs have been investigated for the fabrication of antibacterial packages and protective clothing, for water treatment, as antifouling agents, and for the prevention of microbially influenced corrosion (MIC) (Zheng et al., 2018; Wang et al., 2019a; Bhattacharjee et al., 2019; Firouzjaei et al., 2020; Lu et al., 2019; Kumar et al., 2019; Krishnamurthy et al., 2013). Furthermore, a vast number of studies related to the medical field describe antibacterial materials containing Gr, GO, or rGO for several applications including wound dressing, tissue engineering, and drug delivery and for the prevention of biofilm formation on medical equipment and implantable devices (Ji et al., 2016; Ramasamy and Lee, 2016; Cacaci et al., 2019; Hu et al., 2010).

### Antibacterial Packages and Fabrics

Graphene materials with antibacterial properties can be employed for food packaging as well as in the textile industry (Kumar et al., 2019; Hu et al., 2010; Bhattacharjee et al., 2019). Multiple examples of packaging films made with GMs have been described in the literature. For instance, cross-linked GO with the natural polymer chitosan inhibited the growth of both the Gram-negative *E. coli* and the Gram-positive *B. subtilis* while exhibiting a mechanical strength and thermal stability suitable for food packaging (Grande et al., 2017). In a different study, Ghanem et al. modified GO with hydrophobic poly(4-vinylbenzyl chloride) to facilitate its dispersion into a polystyrene matrix (Ghanem et al., 2020). When compared with unmodified polystyrene, the composite film showed higher thermal stability, better mechanical properties, lower water vapor permeability, and a biocidal effect on pathogenic bacteria.

The addition of GMs to fabrics has been shown to improve antibacterial activity as well as other important properties such as mechanical strength, conductivity, abrasion resistance, UV protection, and flame resistance (Bhattacharjee et al., 2019). Because of these characteristics, textiles modified with GMs, which also restrict gases diffusion, are good candidates for the fabrication of personal protective equipment (PPE). Many studies have described GM-modified fabrics inhibiting bacterial metabolism. For example, pure cotton and cotton/nylon doped with GO, rGO, or rGO combined with antimicrobial chlorinated N-halamine exhibited significant activity against both Gram-positive and Gram-negative bacteria (Pan et al., 2018; Zhao et al., 2013c; Hasani and Montazer, 2017). Self-cleaning wool or cotton modified with a Gr/TiO_2_ nanocomposite also showed high antibacterial activity and could perform photocatalytic removal of contaminants under sunlight (Shirgholami et al., 2016; Stan et al., 2018). Synthetic fabrics including polyester and poly (vinyl alcohol) (PVA) were also modified with GMs. Both polyester doped with rGO/Ag nanocomposites and GO-embedded PVA prevented bacterial growth (Moazami et al., 2016; Hu et al., 2017).

### Water Treatment Membranes and Antifouling

Membranes employed for water treatment and wastewater recycling are highly susceptible to biofouling, which is the formation of damaging biofilm on their surface (Aslam et al., 2017). This phenomenon is a major obstacle for the long-term usage of membranes and is associated with increased cost (Meng et al., 2017; Miura et al., 2007). Numerous studies have successfully combined antimicrobial GMs with polymeric membranes or foams to prevent biofouling (Firouzjaei et al., 2020; Wang et al., 2019a; Perreault et al., 2015a). These composite materials can be employed for different applications such as ultrafiltration, nanofiltration, forward or reverse osmosis desalination, wastewater treatment, as well as radioactive metal recovery from seawater (Zinadini et al., 2014; Choi et al., 2013; Lee et al., 2013; Perreault et al., 2014; Zeng et al., 2016; Guo et al., 2019, 2020; Singh et al., 2018; Mokkapati et al., 2017; Bao et al., 2011). Recent studies describing GM-comprising membranes with antifouling capacity include the development of polysulfone coated with a PDA layer and GO nanosheets (Cheng et al., 2020). This ultrafiltration membrane exhibited strong antibacterial activity against the model bacterium *E. coli*. Graphene-rubber silicone is another example of a composite elastic membrane for water treatment inhibiting bacterial attachment (Jin et al., 2019). More complex antifouling membranes have also been developed such as GO with an Ag-based metal-organic framework (MOF) incorporated in polyethersulfone (PES) (Firouzjaei et al., 2018). This composite material synthesized for forward osmosis showed a strong synergistic interaction between GO and Ag-MOF leading to superior protection against biofouling by *E. coli*. The three studies highlighted above illustrate how GMs can be combined with different polymeric materials to reduce biofouling stymieing membrane-associated applications.

### Water Disinfection via Photocatalysis, Hydrogel Filters, and Aggregates

**Figure 6:**
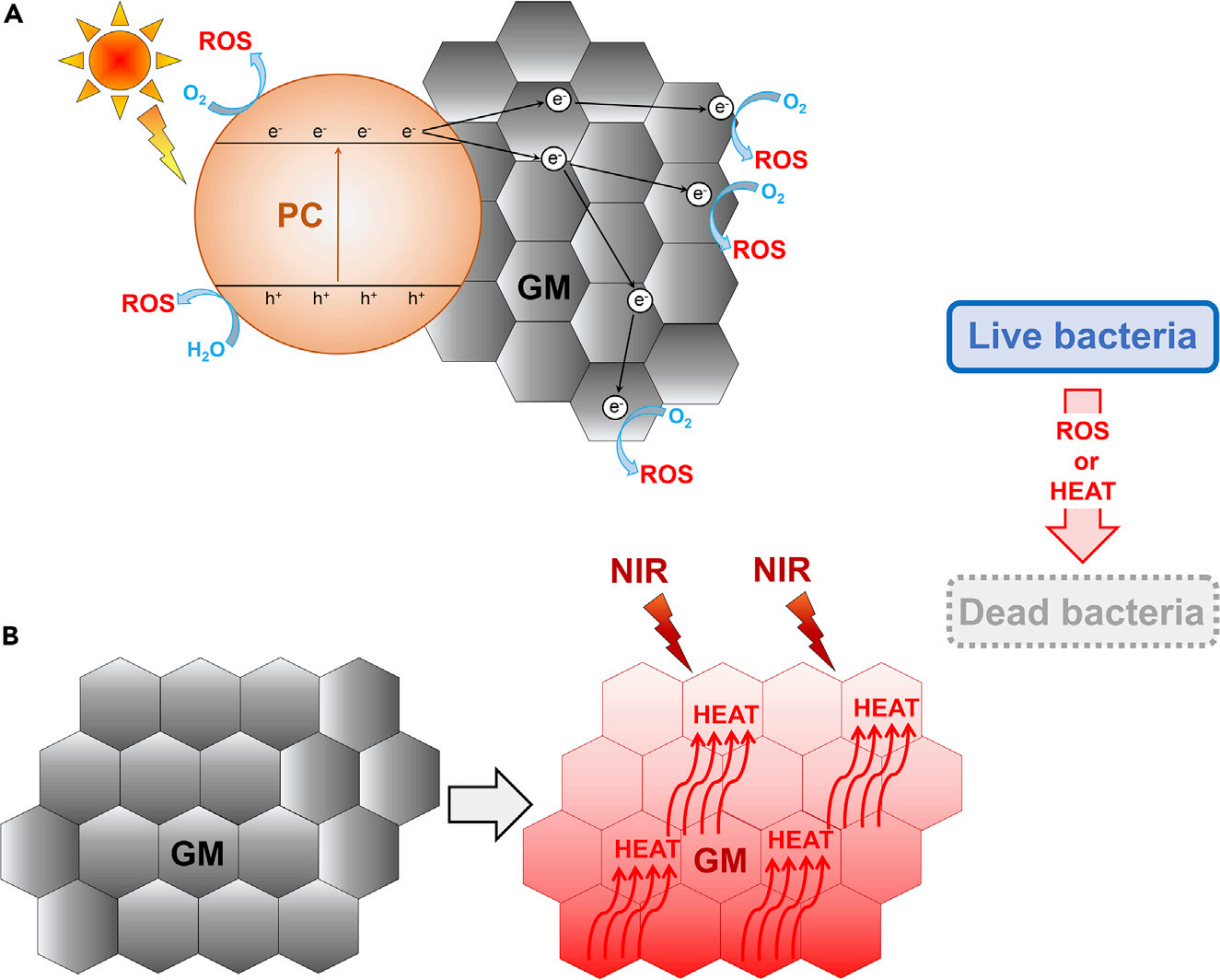
Photocatalytic and Photothermal Disinfection with GMs (A) GMs can be combined with a photocatalyst to accelerate ROS generation and the removal of bacteria under visible light. The main function of GMs in these systems is to serve as the electron acceptor for the photocatalyst (PC) and to facilitate charge separation. (B) Under near-infrared (NIR) radiation, GMs conduct heat and can be employed for photothermal bacterial killing.

Besides membrane-based filters, GMs have been incorporated in other antibacterial devices for water treatment such as photocatalysts, hydrogels, and recyclable aggregates (Wang et al., 2019a). Photocatalytic bacterial killing involves materials that generate ROS upon light irradiation (Gao et al., 2013; Li et al., 2018c). These photocatalysts attach themselves to the bacterial surface where they enter into a charge-separated state after illumination and produce ROS by reducing O_2_ or oxidizing H_2_O molecules (Nosaka and Nosaka, 2017). For this application, GMs are not capable on their own of ROS generation from light because of a small band gap and must be combined with photocatalytic semiconductors (Figure 6) (Avouris, 2010). For instance, a cellulose acetate (CA) support coated with rGO and the photocatalyst graphitic-carbon nitride (g-C_3_N_4_) readily inactivated *E. coli* cells upon illumination and could also remove bacteria from real surface water (Zhao et al., 2016). Reduced graphene oxide probably contributes to this water disinfection system via its own antibacterial properties and by serving as an electron acceptor for g-C_3_N_4_. In this function, rGO accelerates photogenerated charge separation and ROS generation by the photocatalyst (Zhang et al., 2011a). Additionally, multiple studies described photocatalytic water treatment systems where rGO or GO was combined with the metallic photocatalyst TiO_2_ (Wang et al., 2019a). Reduced graphene oxide with TiO_2_, carbon dots and TiO_2_ on rGO, WO_3_ and TiO_2_ on rGO, as well as (Ni(OH)_2_) decorated on GO and TiO_2_ are all examples of photocatalytic systems generating superoxide and/or hydrogen peroxide under light and driving water disinfection (Fernández-Ibáñez et al., 2015; Zeng et al., 2017a, 2017b; Barakat et al., 2020).

Porous filters for water disinfection can be made of GM hydrogels forming three-dimensional foam-like structures (Cong et al., 2014). Multiple examples of antibacterial hydrogels combining GM with metal, such as Ag, Cu, and Ru, have been reported in the literature (Zeng et al., 2015; Chen et al., 2016a; Deng et al., 2017a, 2017b; Sahraei and Ghaemy, 2017; Xue et al., 2016). A potential problem with these materials previously highlighted by Wang et al. is the risk of releasing toxic metal ions in the disinfected water (Wang et al., 2019a). Reduced graphene oxide hydrogels have also been fabricated with other types of antibacterial compounds including tannic acid and a perylene derivative carrying two quaternary ammonium salts (Wang et al., 2019b; Luo et al., 2016).

Another approach for water disinfection with GMs relies on recoverable aggregates. Graphene material nanoparticles can be dispersed in the water to be treated but must be easy to recover to minimize cost and ensure acceptable water quality. For instance, positively charged TiO_2_ nanoparticles aggregated with negatively charged GO in large photocatalytic structures and could be quickly removed by sedimentation from the aqueous solution (Wang et al., 2016). This composite designed for stormwater disinfection deactivated 99.5% of *E. coli* after 90 min of illumination. Another strategy is to develop GM-based composites that can be recuperated via a magnetic field. For example, magnetic rGO was fabricated by microwave irradiation of GO and ferrocene (Gollavelli et al., 2013). These reusable magnetic rGO nanoparticles killed *E. coli* with 100% efficiency and had low toxicity toward zebrafish, which suggests that they could be used for drinking water disinfection. Other magnetic GM-based composites for antimicrobial applications include MnFe_2_O_4_-Gr as well as iron oxide nanoparticles and Ag nanoparticles on GO (Chella et al., 2015; Tian et al., 2014; Zhang et al., 2016; Moosavi et al., 2015).

### Prevention of Microbially Influenced Corrosion

Corrosion of buried metallic pipes is an important economic problem leading to major infrastructure spending. In the United States, 20% of yearly corrosion-related costs are thought to be related to the activity of microbes (Usher et al., 2014). Biocorrosion or MIC involves multiple microbial mechanisms including direct EET with metals acting as solid electron donor, biosynthesis of enzymes attacking metals, production of volatile corrosive molecules, acidity generation, the formation of biological galvanic cells, and differential aeration due to the presence of dense biofilms (Tremblay et al., 2017). Microbially influenced corrosion can be controlled by inhibiting microbial metabolism via different strategies such as cathodic protection, biocide, protective coating, and competitive beneficial bacterial biofilm (Rasheed et al., 2019; Guo et al., 2018). Several studies have investigated bacteria-repulsing and bactericidal GMs for the coating of metallic surfaces to prevent MIC. For instance, MIC was reduced 40 times when a nickel surface was coated by CVD with pristine Gr (Krishnamurthy et al., 2013). In fact, Gr coating was shown to provide a 10- to 100-fold superior protection against MIC compared with two widely used surface-protecting polymers, parylene-C and polyurethane (PU) (Krishnamurthy et al., 2015). Alternatively, GO has been combined with the polymer epoxy acrylate-PU, which improved GO distribution at the surface of carbon steel (Ahmadi and Ahmad, 2019). The GO-containing composite coating exhibited strong contact killing for both Gram-negative and Gram-positive bacteria. Another important characteristic of GMs for the prevention of biocorrosion is their low permeability related to the small pore size of the carbon lattice (Amorim et al., 2007; Malhotra et al., 2020). Because of this attribute, GM-based coatings not only block direct contacts between microbes and metallic surfaces but may also prevent the leaching of small molecules such as metallic ions that could be employed by the metabolism of corrosive bacteria.

### Antibacterial Graphene for Therapeutic Applications

Graphene materials in the medical field have been studied extensively for therapeutic applications such as drug delivery systems, tissue engineering scaffolds, and wound sterilization (Bai et al., 2019; Wu et al., 2017; Zhang et al., 2017a; Ghawanmeh et al., 2019). Antibacterial GMs have many suitable characteristics for a large utilization in biomedicine including low cost and an easy synthesis process. Furthermore, GMs can easily be combined with other compounds to form synergistic composites and their antibacterial activity involves multiple mechanisms acting simultaneously, which renders difficult the development of bacterial resistance often observed with antibiotics (Yousefi et al., 2017).

On the other hand, Xia et al. (2019) highlighted two major issues with GMs for clinical utilization: cytotoxicity toward animal cells and biomacromolecule absorption (Xia et al., 2019). Conflicting studies have been published on the cytotoxicity of Gr-containing nanomaterials for eukaryotic cells and animal models (Lu et al., 2019). Some reports described little or no toxicity of GMs, whereas others detailed important dose-dependent cytotoxicity related to the generation of oxidative stress (Wu et al., 2018; Akhavan et al., 2015; Gollavelli and Ling, 2012; Zhang et al., 2012; Xu et al., 2015). For instance, GMs have exhibited cytotoxic effects on both human erythrocytes and skin fibroblasts (Liao et al., 2011). Eukaryotic cells are highly sensitive to some of the mechanisms responsible for the antibacterial activity of GMs including membrane stress and oxidative stress (Cooper and McNeil, 2015; Pizzino et al., 2017). Thus, it is not surprising that GMs designed for inhibiting the growth of a broad range of microbes via wide and untargeted mechanisms would also be toxic for animal cells. Dosage is probably critical in determining if GMs will be toxic for eukaryotic cells and must be investigated with care for the development of therapeutic applications.

The second issue with antibacterial GMs for application in physiological fluids is their capacity to readily absorb proteins and other biomacromolecules on their surface (Xia et al., 2019; Castagnola et al., 2018; Bhattacharya et al., 2016; Duan et al., 2015a). The formation of a biomacromolecule layer or corona changes the physicochemical properties of GMs and creates a physical barrier preventing direct contact with bacteria. Because of this phenomenon, the antibacterial activity of GMs will be dampened over time.

### Antibiofilm Agent for Medical Equipment and Implantable Devices

Biofilm formation by pathogenic bacteria on medical equipment and implantable devices causes material damage and may lead to clinical complications as well as chronic infections (Campoccia et al., 2013) Medical and implantable devices that can be colonized by biofilm include heart valves, prostheses, endotracheal tubes, catheters, contact lenses, implants, and surgical instruments (Ramasamy and Lee, 2016). Because of their antibacterial properties, GMs have been explored as potential coatings for medical equipment and implantable devices (Kumar and Chatterjee, 2016). Besides standard coating approaches with antibacterial GMs or GM composites, photothermal therapy relying on the conversion of light into heat is another promising strategy for bacteria killing and the sterilization of medical equipment surfaces (Yang et al., 2012). The capacity of GMs to conduct heat under certain types of light irradiation is an important characteristic that has raised a lot of attention for the removal of biofilms (Figure 6B) (Nafiujjaman and Nurunnabi, 2019). Photothermal therapy is being developed because it has several benefits over biocidal agents normally used to prevent biofilm formation including a broad bactericidal range, no development of microbial resistance, and no toxic effect on human health (Xu et al., 2019b). Examples of GMs developed for photothermal bactericidal therapy include a near-infrared (NIR) laser-excited film made with GO and PAH, an NIR irradiation-excited polyelectrolyte-rGO composite on a quartz substrate, and an ultrafiltration membrane made of rGO and bacterial nanocellulose also activated by NIR irradiation (Hui et al., 2015; Kurapati et al., 2016; Jiang et al., 2019; Zou et al., 2020).

## Perspective

In summary, adjustable physicochemical properties and environmental factors determine if Gr, GO, or rGO will promote bacterial metabolism and growth or if they will act as antibacterial materials. For bioelectrochemical applications, GMs have shown promising performance as electrode coatings because they combined suitable electrical and physical characteristics with high affinity for bacterial cells leading to the formation of healthy electroactive biofilms (Aryal et al., 2017a; ElMekawy et al., 2017). Bioelectrochemical reactors are operated under anaerobic conditions, which seems to be an important reason why GMs do not inhibit bacterial growth in these systems. As a matter of fact, the toxicity of GMs toward bacteria is partly explained by its capacity to engender oxidative stress with ROS generated from molecular oxygen (Perreault et al., 2015a; Zou et al., 2016). This simple observation raises possible concerns for the long-term stability of several antibacterial applications of GMs in environments where oxygen is absent or quickly depleted such as coating for pipes affected by anaerobic MIC or antifouling agent in anaerobic membrane bioreactor for wastewater treatment (Li et al., 2018d; Gao et al., 2010).

Another aspect of laboratory research investigating antibacterial GMs that possibly does not reflect what happens in actual environments is that many viability tests have only been performed with pure cultures of model bacteria such as *E. coli*, *P. aeruginosa*, *B. subtilis*, and *S. aureus* (Firouzjaei et al., 2020; Zheng et al., 2018; Xia et al., 2019). Metallic structures susceptible to biocorrosion, aqueous environments, the surface of medical devices or of other objects, as well as wounds are often populated by mixed microbial communities comprising strains and species differing from the laboratory-used ones that may not be affected by the antibacterial activity of GMs (Buch et al., 2019; Stacy et al., 2016; Lee et al., 2014; Bruno et al., 2018; Coad et al., 2016; Rajala et al., 2015). Based on these observations, future research would benefit from studying GMs for both long-term antibacterial and bacterial growth-promoting applications in real-life conditions and not in controlled laboratory conditions.

In the case of therapeutic applications such as drug delivery, tissue engineering, and wound dressing with antibacterial GMs, there is an additional challenge besides efficacy related to safety. Several studies demonstrated the toxicity of several GMs on mammalian cells and in animal models (Guo and Mei, 2014; Fadeel et al., 2018). Although further research and better standardization between studies are needed to have a comprehensive portrait of the real cytotoxicity of GMs, questions must be asked about the ethical issues raised by multiplying experiments in animal models with a material that may never be safe and/or efficient enough for therapeutic applications.
